# Supplementary material for: Effect of sulfur on sound velocity of liquid iron under Martian core conditions
Source: Nat Commun. 2020 May 13;11:1954. doi: 10.1038/s41467-020-15755-2 (PMC7220916; doi:10.1038/s41467-020-15755-2)
Supplement: Supplementary file 1 — Supplementary Information [file 41467_2020_15755_MOESM1_ESM.pdf]

Supplementary Information for

Effect of sulfur on sound velocity of liquid iron under Martian core conditions

by Nishida *et al*.

Supplementary Notes

Supplementary Note 1
Comparison with previous measurements

We compare the present velocity data with those of liquid Fe-Ni-S recently obtained[1] using a similar method (Supplementary Fig. 2). Indeed, both data set are consistent with each other when we consider larger errors in velocity in ref. 1, in the sense that higher sulfur content reduces V_P below ~10 GPa.

The earlier inelastic X-ray scattering (IXS) study[2] in a diamond-anvil cell reported the V_P of liquid $(Fe,Ni)_{75}S_{25}$ at ≥10 GPa. The present ultrasonic V_P in liquid $Fe_{80}S_{20}$ is in good agreement with these IXS measurements[2], although the former is a little lower around 10 GPa (Supplementary Fig. 3). These results suggest that difference in the frequency range between the ultrasonic and IXS measurements does not result in different V_P obtained.

Our V_P of liquid Fe is consistent with that measured by ref. 3 but a little bit lower than that by ref. 4 (Supplementary Fig. 1a). This is possibly due to a contamination effect on their V_P as mentioned in ref. 4. The pressure dependence of V_P in liquid Fe observed in this study is very similar to that estimated from the equation of state (EoS) of liquid Fe (ref. 5), which has been commonly used as a reference, but our absolute value is slightly higher than that by the EoS at ≥ 10 GPa. The V_P of liquids $Fe_{80}S_{20}$ and $Fe_{57}S_{43}$ obtained here are in good agreement with those determined by ref. 4 (Supplementary Figs. 1b, c).

Supplementary Note 2
Cause of the change in dV_P/dP of liquid Fe-S around 10 GPa.

We argue in the main text that the dV_P/dP slope for liquid $Fe_{80}S_{20}$ changes around 10 GPa (Fig. 2a) as a consequence of spin crossover in the FeS-like portion in liquid. Here we discuss it in more detail.

Liquid structure is considered to be often relevant to the structure of a solid phase before melting occurs. Where there is a large structural change between solid phases, there would be a liquid-liquid phase transition and a large change in physical property. Solid Fe transforms from bcc into fcc around 5 GPa just below the melting temperature. X-ray structural study reported that liquid Fe also changes its structure from the bcc-like to the mixed bcc + fcc structure[6]. It affects the V_P of liquid Fe to a minor extent because the volume difference between the bcc and fcc structures is small (Fig. 2b), and the structural change in liquid occurs in a relatively wide pressure range. Indeed, we did not observe any significant changes in V_P around 5 GPa.

Fe_3S is an intermediate compound formed at >20 GPa and undergoes both magnetic and spin transitions between 15 to 21 GPa[7]. Solid Fe_3S changes its volume little across these transitions in a broad pressure range[8], and the compression curve of Fe_3S is very similar to that of Fe[9,10] (Fig. 2b). Therefore, the spin crossover in Fe_3S is unlikely to be relevant to the change in dV_P/dP for liquid $Fe_{80}S_{20}$ around 10 GPa (Fig. 2a). The high dV_P/dP slope of liquid $Fe_{80}S_{20}$ observed below 10 GPa is not consistent with the compression curve of solid Fe_3S, suggesting that the structure of liquid $Fe_{80}S_{20}$ is not similar to that of solid Fe_3S.

Indeed, an anomalous behavior in liquid $Fe_{79.3}Ni_{4.4}S_{16.3}$, the increase in sound velocity with increasing temperature, was reported at 1 bar[11]. Such anomalous behavior is also known in liquid H_2O; it is found in an inhomogeneous mixed state between sparse (low-*P* phase) and dense structures (high-*P* phase)[12]. As well in the Se-Te system[13,14], an inhomogeneous mixture of 2-hold semiconducting Se-like structure and 3-hold metallic Te-like structure causes the similar behavior; the velocity increases at higher temperatures.

Supplementary Note 3

Liquidus and solidus temperature in the Fe-FeS system

We examined whether the Mars has a solid core by comparing the liquidus temperatures in the Fe-FeS system and possible temperature profiles in the Martian core. For pure Fe, the present experimental data are consistent with relatively low melting curves[15,16] (Supplementary Fig. 9a), and here we employ the one by Boehler *et al.*[15] For the FeS end-member, the melting curve exhibits a steep dT/dP slope at low pressures[17,18] (Supplementary Fig. 9b). At higher pressure range, the one reported by ref. 19 is consistent with both our data obtained around 20 GPa and shock-compression data[20], but does not agree with the low pressure experiments[17,18]. It is therefore likely that the melting curve of FeS changes its dT/dP slope around 5 GPa. Indeed, it could be because of spin crossover in liquid FeS, which is suggested from the present velocity measurements.

The present experiments demonstrated that the liquidus temperature of $Fe_{80}S_{20}$ increases linearly with pressure with the maximum at 18 GPa where Fe_3S is formed as an intermediate compound and the system becomes Fe-Fe_3S eutectic (Supplementary Fig. 9c). The liquidus curve of $Fe_{80}S_{20}$ is extrapolated to higher pressures by using a eutectic temperature at 36.8 GPa where $Fe_{80}S_{20}$ is a eutectic liquid composition[21]. The liquidus temperature of $Fe_{57}S_{43}$ decreases to 1.5 GPa where $Fe_{57}S_{43}$ is a eutectic composition[22] and then increases rapidly to 5 GPa, likely due to the effect of spin

crossover as we argued for the FeS end-member above. Subsequently the slope changes, and the liquidus curve of $Fe_{57}S_{43}$ becomes parallel to that of FeS (Supplementary Fig. 9d).

These liquidus curves under the Martian core pressure range are illustrated in Supplementary Fig. 12, in comparison with estimated Mars' core temperatures[23,24]. It indicates that if the core composition is between $Fe_{80}S_{20}$ and $Fe_{57}S_{43}$, the core should be fully molten (no solid core), while solid core could exist when the Martian core composition is between Fe and $Fe_{80}S_{20}$ or between $Fe_{57}S_{43}$ and FeS.b

Supplementary Figures

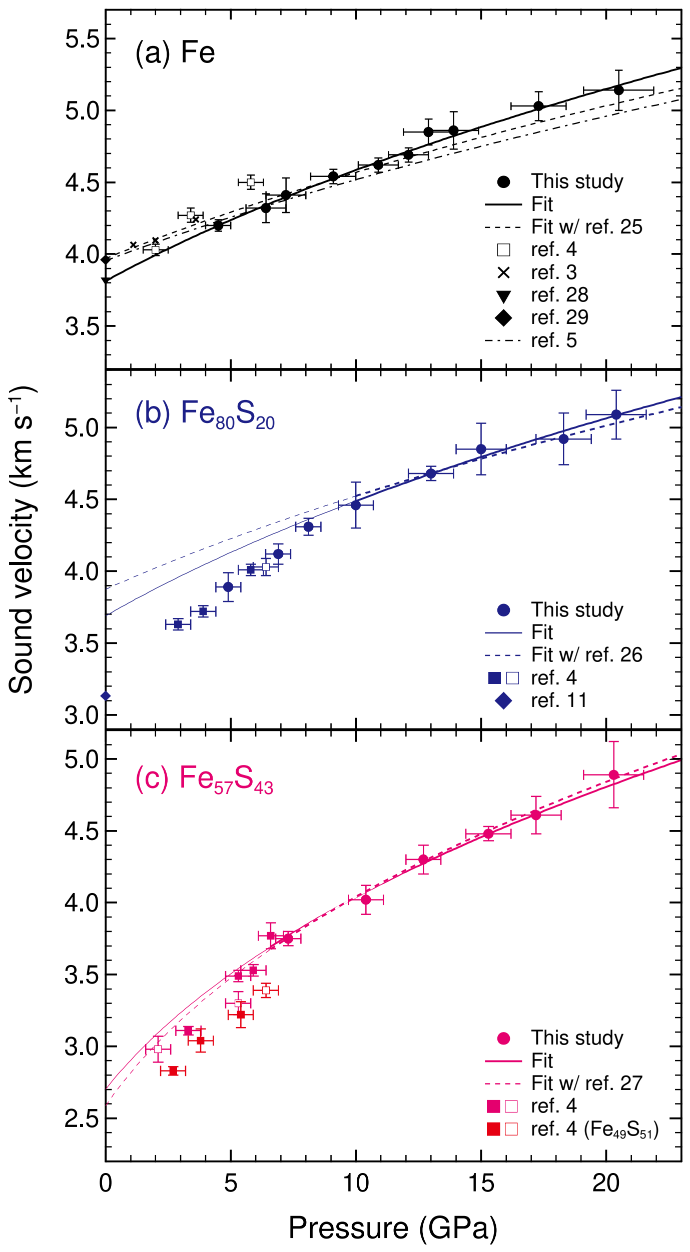

Supplementary Figure 1 | Sound velocity in liquid Fe and Fe-S alloys at high pressure. Solid circles show the present ultrasonic V_P data. Solid and dashed curves are obtained by fitting without and with using previous shock-wave data for Fe (ref. 25), $Fe_{80}S_{20}$ (ref. 26) and FeS (ref. 27), considering the effect of spin crossover in the FeS-like portion in liquid (see text). **a,** Liquid Fe. Open squares[4] and crosses[3] denote ultrasonic V_P from previous studies. Solid reverse triangle[28] and diamond[29] show 1-bar data. Dash-dotted curve is calculated from the EOS[5]. **b,** Liquid $Fe_{80}S_{20}$. Open and solid squares represent ultrasonic V_P from ref. 4. Solid diamond denotes the ultrasonic V_P of liquid $Fe_{79.3}Ni_{4.4}S_{16.3}$ at 1 bar[11]. **c,** Liquid $Fe_{57}S_{43}$. Open and solid squares show previous ultrasonic V_P data for liquid $Fe_{57}S_{43}$ (pink) and $Fe_{49}S_{51}$ (red), respectively[4].

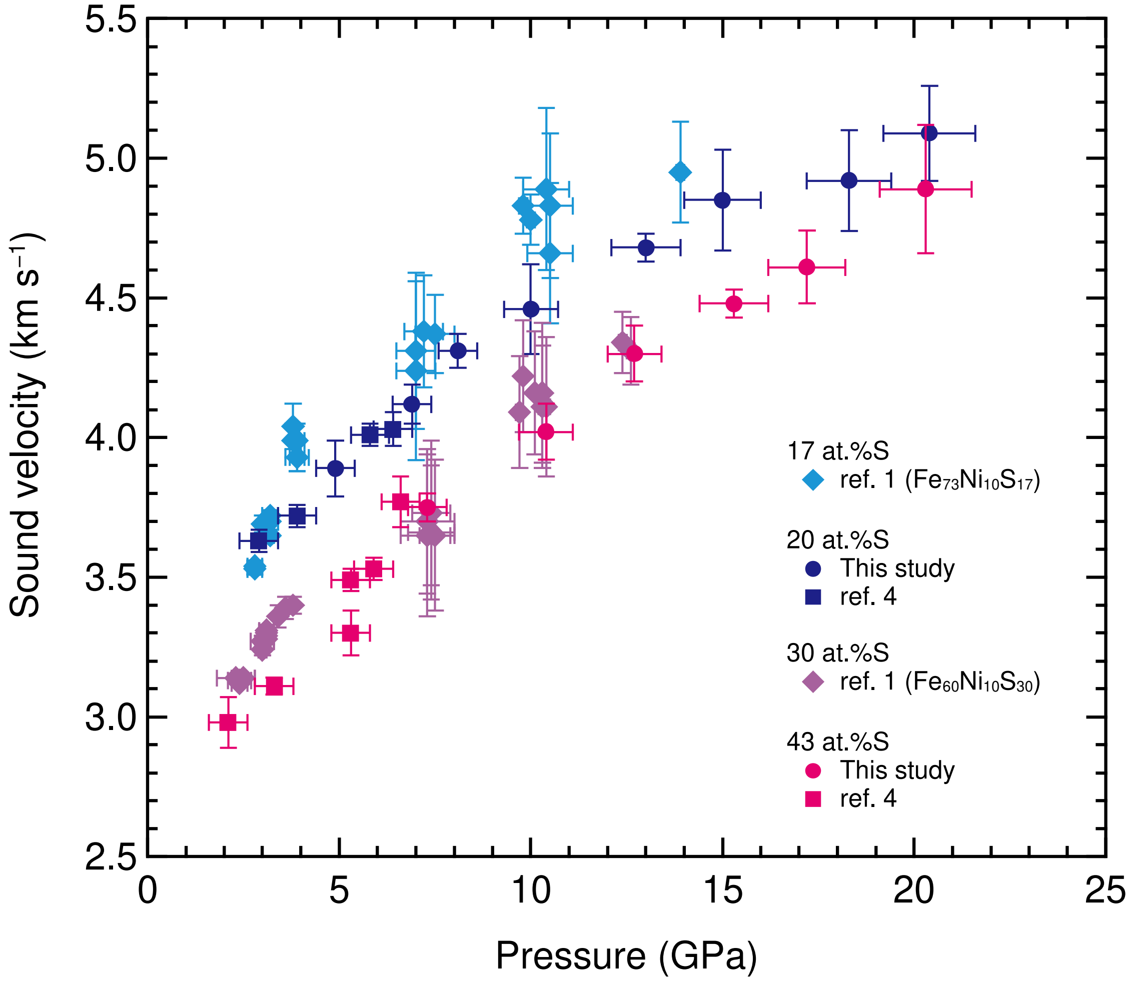

Supplementary Figure 2 | Effect of Ni on V_P in liquid Fe-S at high pressure. Data for nickel-bearing liquids are from Terasaki *et al.*[1]

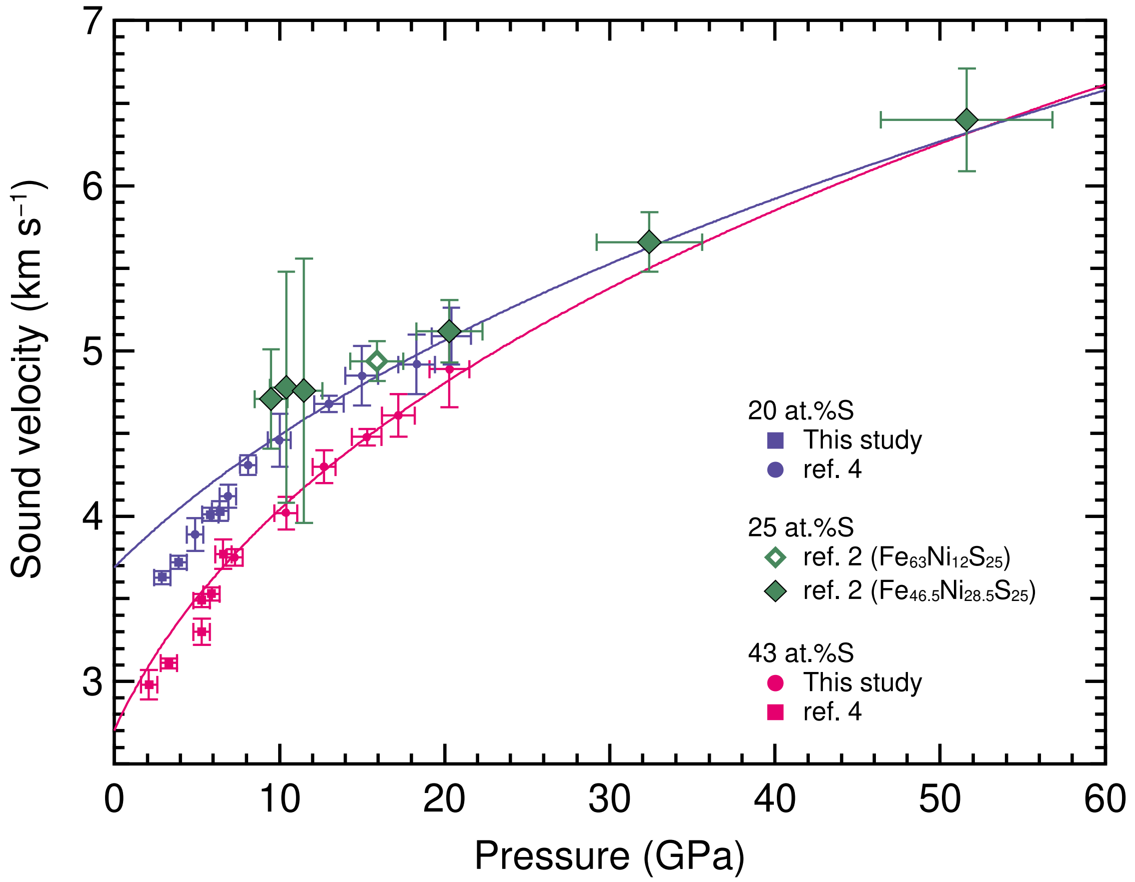

Supplementary Figure 3 | Comparison of V_P between the present ultrasonic (20 and 43 at.% S) and previous IXS measurements (25 at.% S). The IXS data are from Kawaguchi *et al.*[2]

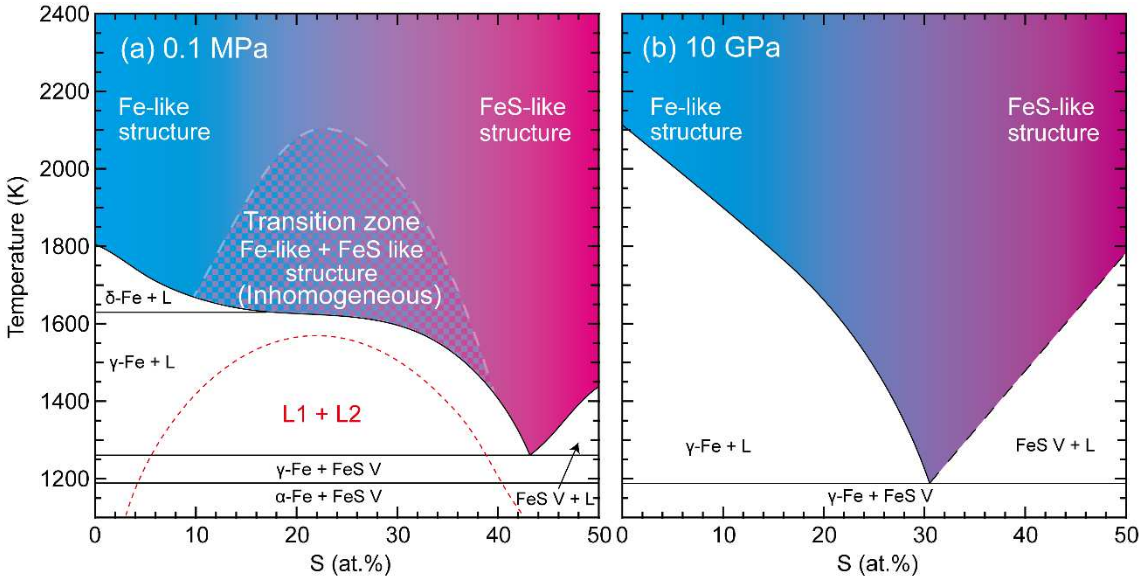

Supplementary Figure 4 | Phase diagrams of the Fe-FeS system a, at 0.1 MPa modified after ref. 30; **b,** at 10 GPa modified after ref. 31.

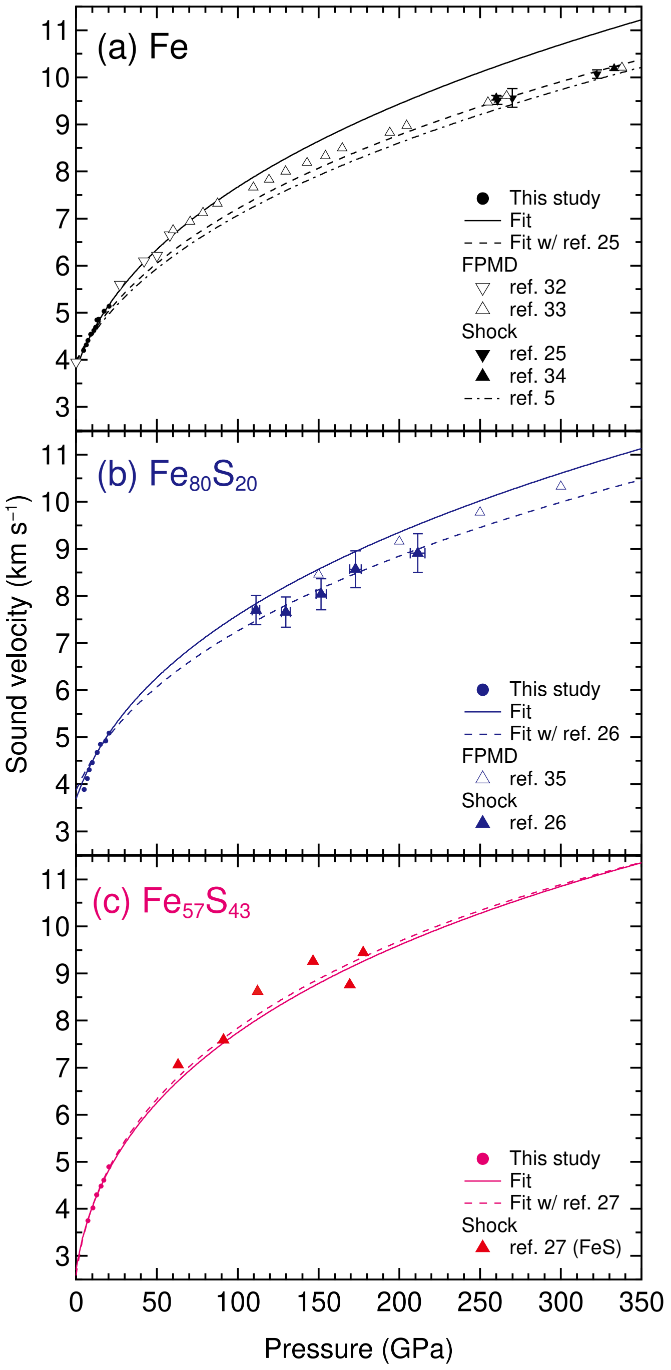

Supplementary Figure 5 | Comparison of present P–V_P curve with previous theoretical calculations and shock-wave experiments. Filled circles are from this study. Solid and broken curves are fitting results without and with shock data, respectively, for Fe (ref. 25), $Fe_{80}S_{20}$ (ref. 26) and FeS (ref. 27). **a,** Liquid Fe. Open reverse triangles, dynamic first-principles molecular dynamics (FPMD) simulations[32]; open normal triangles, static FPMD[33]; filled reverse[25] and normal[34] triangles, shock compression. Dash-dotted curve is based on EOS[5]. **b**, Liquid $Fe_{80}S_{20}$. Open triangles, static FPMD[35]; filled triangles, shock data[26]. **c**, Liquid $Fe_{57}S_{43}$. Filled triangles, shock compression[27] for FeS.

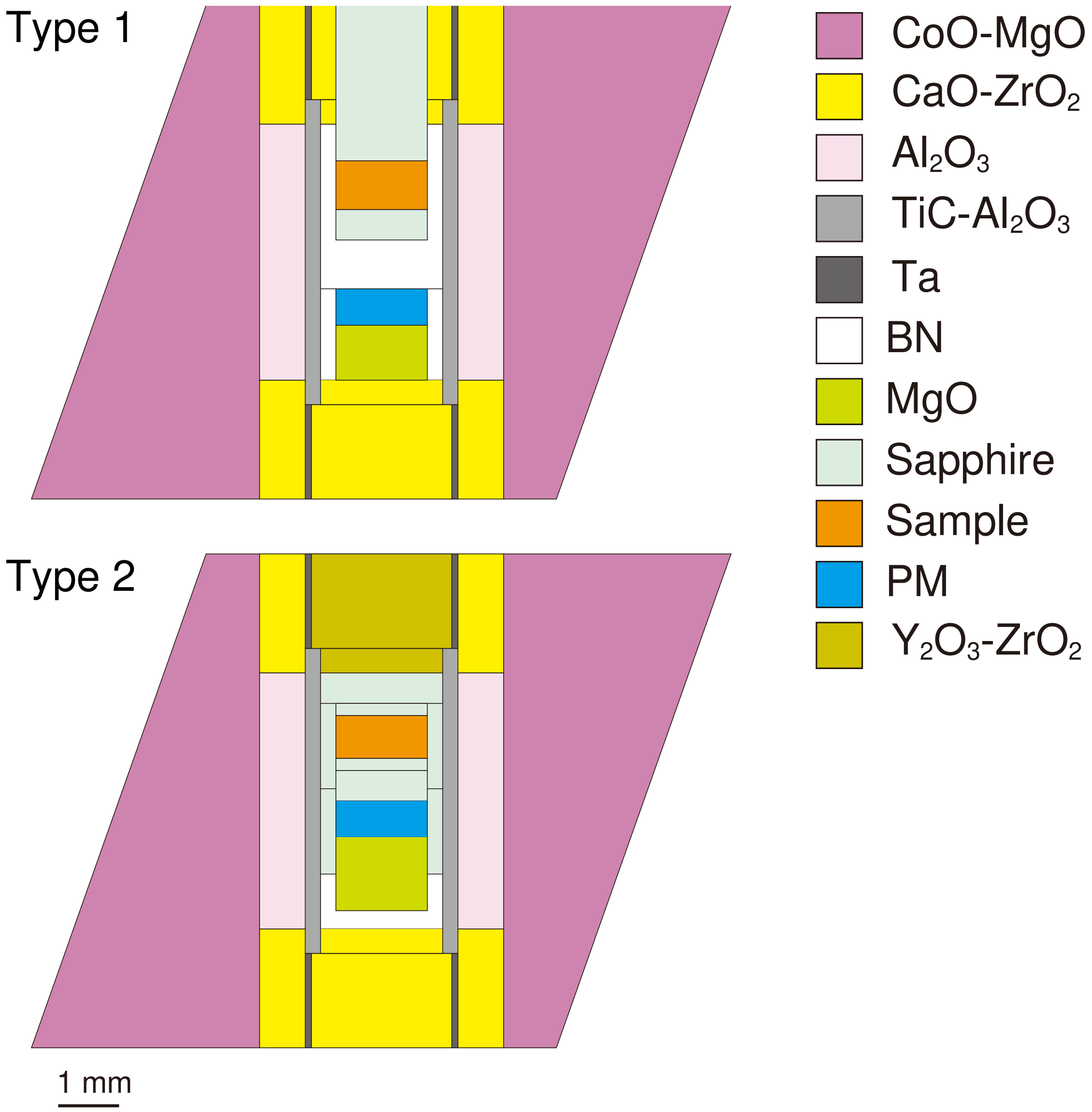

Supplementary Figure 6 | Cross sections of cell assemblies.

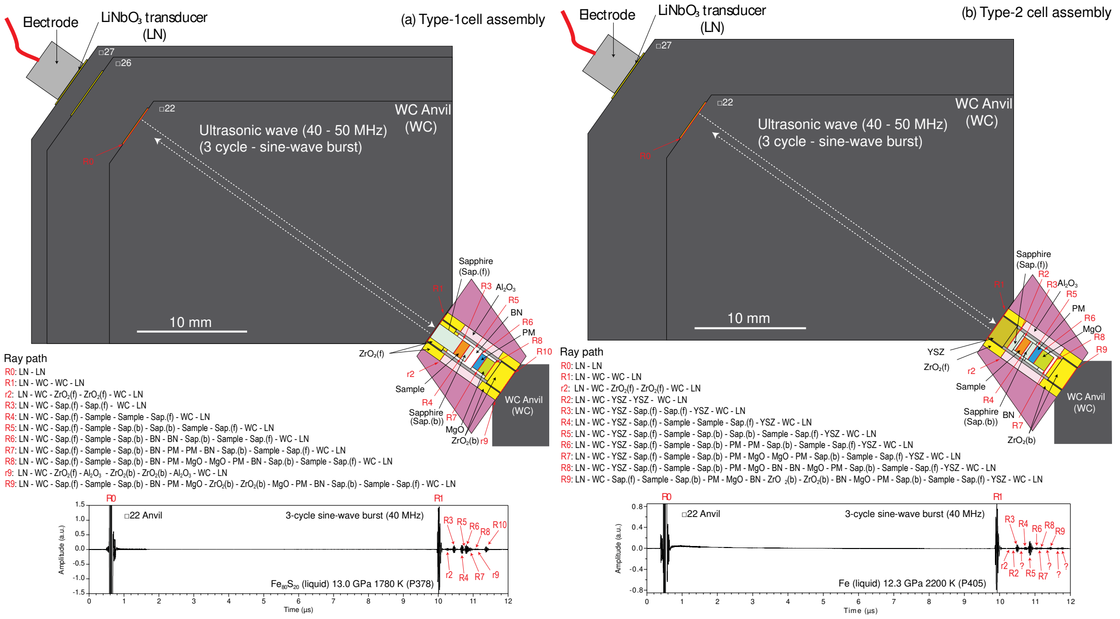

Supplementary Figure 7 | Deployment diagram of cell assembly for ultrasonic measurements (before compression), and examples of ultrasonic echoes and their ray paths. Red lines (R1–R10 or R1–R9) in the cell show reflections that correspond to echo signals R1–R9 in the waveform diagram. **a,** Type-1 cell at 13.0 GPa and 1780 K (Run# P378) for the measurements of liquid $Fe_{80}S_{20}$. **b,** Type-2 cell at 12.3 GPa and 2200 K (Run# P405) for liquid Fe. Some unidentified echoes may be attributed to multiple reflections. Note that the cell size changed upon compression. See Fig. 1 for an enlarged waveform diagram.

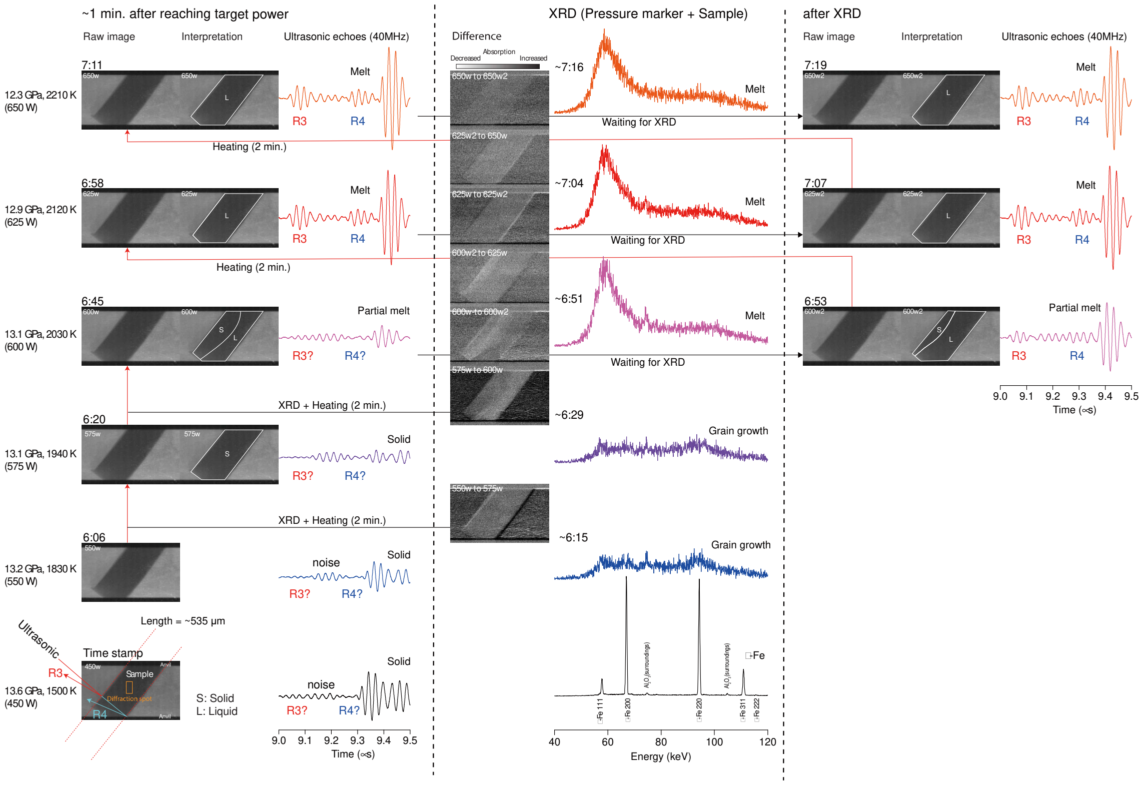

Supplementary Figure 8 | Data acquisition flow in run #P407. See Methods for details. Time is given in each panel. The image next to the XRD pattern shows the difference in radiographic image between the first and the second collections, indicating the distributions of molten and solid parts in partially molten sample.

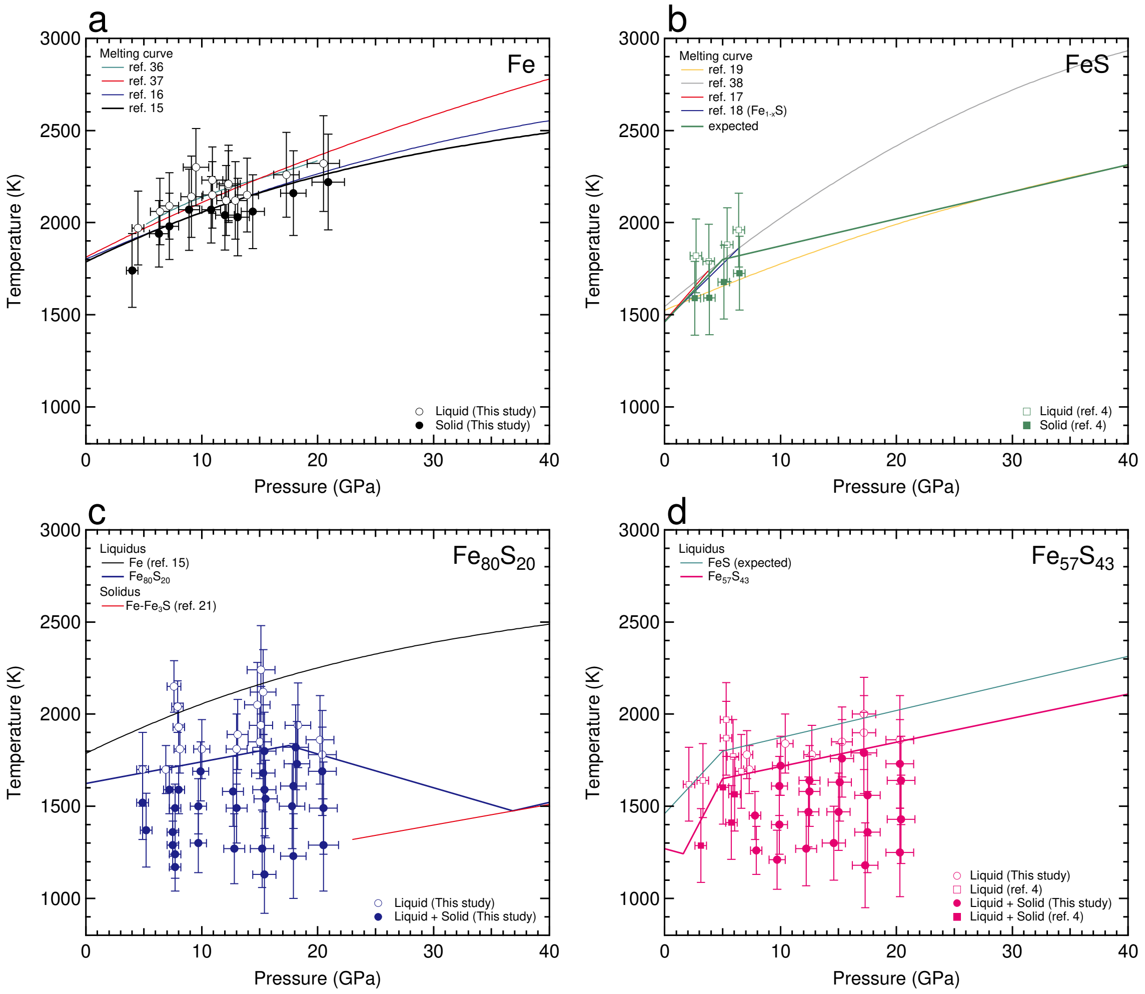

Supplementary Figure 9 | Liquidus curves in the Fe-FeS system. Open symbols denote completely molten state found in the present experiments, while solid symbols show solid or partially molten state. **a,** Fe. Green[36], red[37], black[15] and blue[16] curves from previous experiments. **b,** FeS. Yellow[19], gray[38], red[17] curves were obtained for FeS and blue[18] for pyrrhotite. Spin crossover in liquid FeS likely changes the slope (green curve), which explains both our data and earlier results obtained at higher pressures. **c,** $Fe_{80}S_{20}$. Blue line shows the liquidus temperature estimated in this study. The results by Stewart *et al.*[21] is given by red line. **d,** $Fe_{57}S_{43}$. Pink line indicates the liquidus temperature determined in this study.

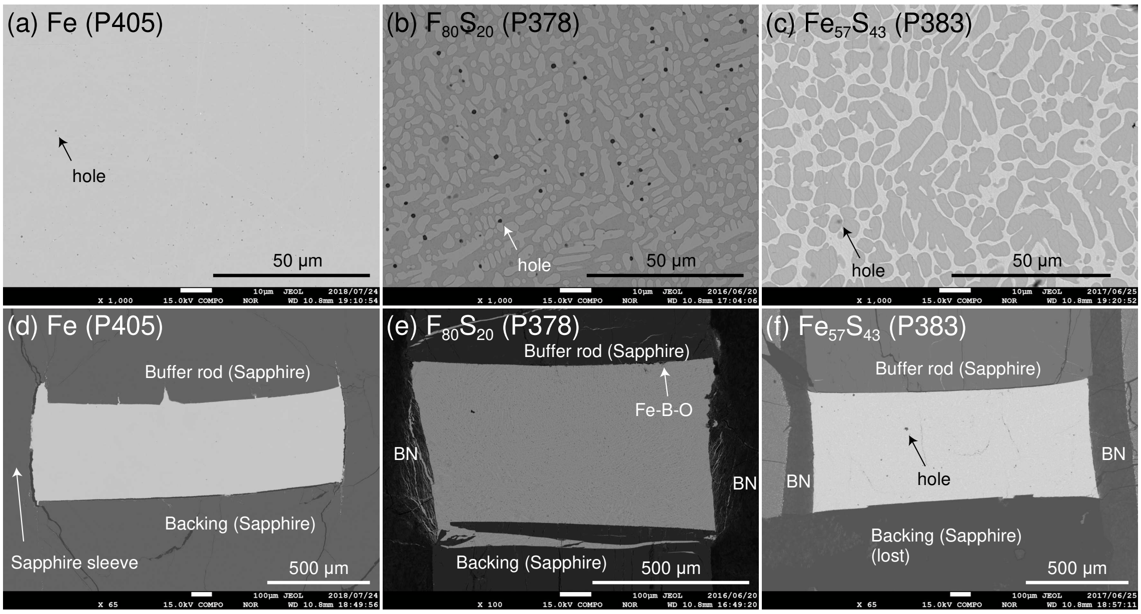

Supplementary Figure 10 | Backscattered electron images of run products. a, d, quenched liquid Fe (run #P405); **b, e,** quenched liquid $Fe_{80}S_{20}$ (run #P378) consisting of Fe (light gray) and eutectic matrix (dark gray); **c, f,** quenched liquid $Fe_{57}S_{43}$ (run #P383) showing a mixture of FeS (dark gray) and eutectic matrix (light gray). The dark spots represent holes. Small oxide crystals were found in samples near the container and the buffer rod.

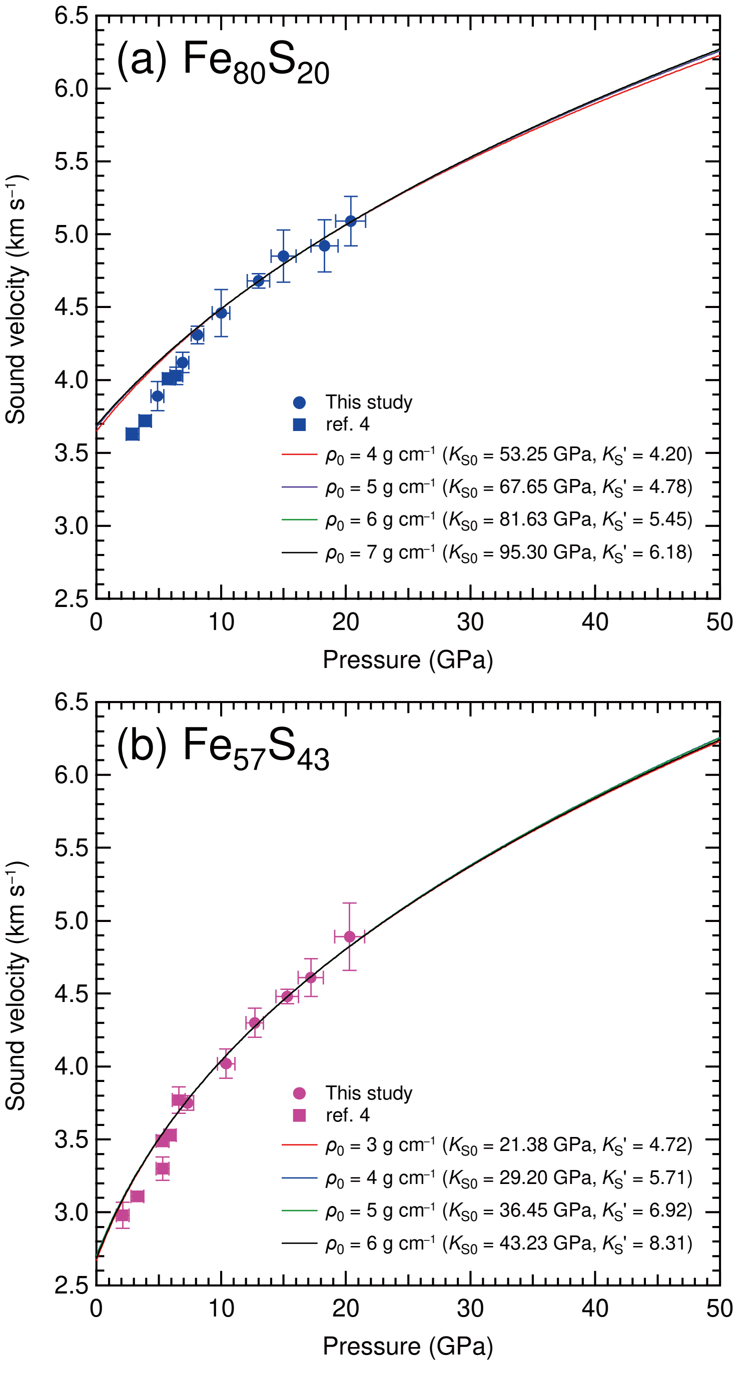

Supplementary Figure 11 | Effect of the choice of ρ_0 on the extrapolations of V_P to higher pressures. a, $Fe_{80}S_{20}$. **b,** $Fe_{57}S_{43}$.

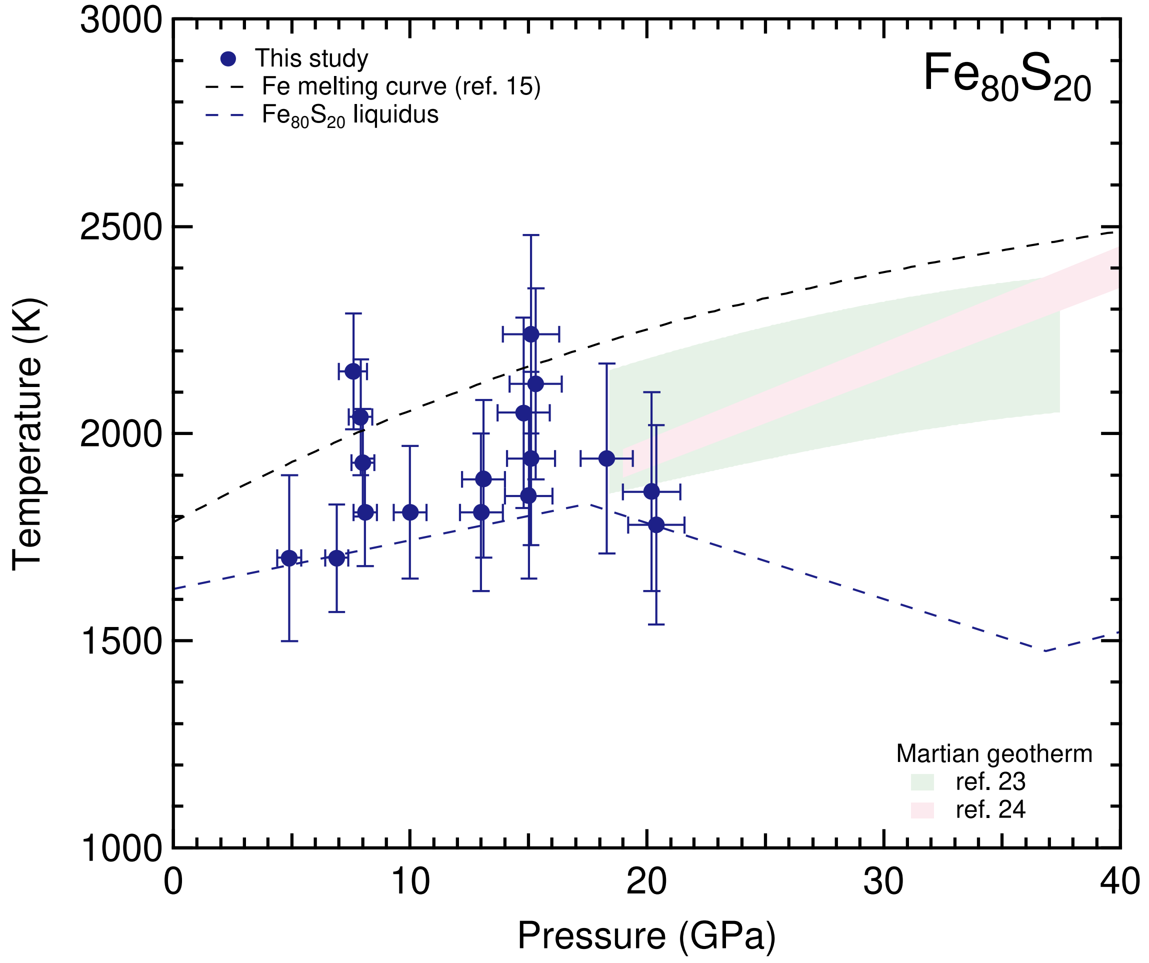

Supplementary Figure 12 | *P-T* for present liquid $Fe_{80}S_{20}$ measurements in comparison to proposed Martian core conditions. Blue solid circles denote conditions for liquid $Fe_{80}S_{20}$ measurements in this study. Pink and green bands show Mars' core temperature models, ref. 23 and ref. 24, respectively. Liquidus curves are from Supplementary Fig. 9.

Supplementary Tables

Supplementary Table 1 | Experimental conditions and results

Label	Run #	Cell type	Pressure (GPa)	Temperature (K)	Sound velocity (km s^{-1})	Chemical compositions				
						Fe (wt%)	S (wt%)	O (wt%)	Total (wt%)	Atomic ratio
Fe	P406	2	4.5(5)[a]	1970(200)[a]	4.20(4)	100.1	-	n.d.	100.1	Fe
	M2412	2	6.4(8)[b]	2060(180)[c]	4.32(10)	100.0	-	0.2	100.2	$Fe_{99.4}O_{0.6}$
	M2414	2	7.2(8)[b]	2090(180)[c]	4.41(12)	100.6	-	0.2	100.8	$Fe_{99.4}O_{0.6}$
	P403	2	9.1(9)[b]	2140(220)[c]	4.54(5)	100.7	-	0.2	100.9	$Fe_{99.4}O_{0.6}$
			9.5(11)[b]	2300(210)[c]	4.53(5)		-			
	P393	2	10.9(8)[b]	2150(180)[c]	4.62(5)	TiC contaminated during cooling				
			10.9(9)[b]	2230(180)[c]	4.62(5)		-			
	P405	2	12.1(8)[d]	2120(190)[c]	4.69(5)	100.1	-	n.d.	100.1	Fe
			12.3(8)[b]	2200(190)[c]	4.68(5)		-			
	P407	2	12.9(10)[b]	2120(210)[c]	4.85(5)	101.2	-	n.d.	101.2	Fe
			12.3(10)[b]	2210(210)[c]	4.84(9)		-			
	M2321	2	13.9(10)[b]	2150(200)[c]	4.86(13)	99.8	-	0.2	100.0	$Fe_{99.3}O_{0.7}$
	M2415	2	17.3(11)[b]	2260(230)[c]	5.03(10)	TiC contaminated				
	M2413	2	20.5(14)[b]	2320(260)[c]	5.14(14)	TiC contaminated				
$Fe_{80}S_{20}$	P370	1	4.9(5)[a]	1700(200)[a]	3.89(10)	86.8	11.8	0.2	98.8	$Fe_{80.4}S_{19.0}O_{0.6}$
	P379	1	6.9(5)	1700(130)	4.12(7)	86.6	12.1	0.1	98.9	$Fe_{80.1}S_{19.4}O_{0.5}$
	P401	2	8.1(5)	1810(130)	4.31(6)	88.8	10.9	0.2	99.8	$Fe_{81.9}S_{17.5}O_{0.6}$
			8.0(5)	1930(130)	4.30(7)					
			7.9(5)[b]	2040(140)[c]	4.30(7)					

			7.6(5)[b]	2150(140)[c]	4.29(7)					
	M2025	1	10.0(7)	1810(160)	4.46(16)	88.4	10.6	0.4	99.3	$Fe_{81.7}S_{17.1}O_{1.2}$
	P378	1	13.0(9)	1780(190)	4.68(5)	87.0	11.4	0.2	98.6	$Fe_{80.9}S_{18.5}O_{0.6}$
			13.1(9)	1890(190)	4.68(5)					
	P404	2	15.0(10)	1850(200)	4.85(18)	TiC contaminated during cooling				
			15.1(10)	1940(210)	4.86(17)					
			14.8(11)[b]	2050(230)[c]	4.86(17)					
			15.3(11)[b]	2120(230)[c]	4.85(17)					
			15.1(12)[b]	2240(240)[c]	4.84(17)					
	M1966	1	18.3(11)	1940(230)	4.92(18)	88.8	10.9	0.3	100.0	$Fe_{81.5}S_{17.5}O_{1.0}$
	M2232	1	20.4(12)	1780(240)	5.09(17)	88.2	11.6	0.3	100.1	$Fe_{80.7}S_{18.5}O_{0.8}$
			20.2(12)	1860(240)	5.09(16)					
$Fe_{57}S_{43}$	M2231	1	7.3(5)	1700(130)	3.75(5)	65.7	34.0	0.2	100.0	$Fe_{52.3}S_{47.0}O_{0.7}$
			7.1(5)	1780(130)	3.75(8)					
	M2229	1	10.4(7)	1840(160)	4.02(10)	66.1	33.4	0.5	100.1	$Fe_{52.4}S_{46.1}O_{1.5}$
	P384	1	12.7(7)	1780(160)	4.30(10)	68.8	30.8	0.5	100.1	$Fe_{55.4}S_{43.2}O_{1.4}$
	P383	1	15.3(9)	1850(190)	4.48(5)	68.4	31.3	0.5	100.1	$Fe_{54.9}S_{43.7}O_{1.4}$
	M2163	1	17.2(10)	1900(200)	4.61(13)	67.5	32.1	0.5	100.0	$Fe_{54.0}S_{44.7}O_{1.3}$
			17.2(10)	2000(200)	4.60(13)					
	M2162	1	20.3(12)	1860(240)	4.89(23)	68.1	31.4	0.6	100.1	$Fe_{54.6}S_{43.9}O_{1.6}$

Note: Numbers in parentheses represent error in the last digits.

[a]Pressure and temperature were determined by MgO + h-BN pressure standards.

[b]Pressure was determined by the MgO standard with temperature estimated.

[c]Temperature was estimated by extrapolation of power-temperature relationship.

Supplementary Table 2 | Chemical composition of contaminated samples

Element (wt%)	M2415	M2413
C	0.9(1)	0.5(1)
O	0.2(1)	0.1(1)
Al	0.1(0)	0.8(1)
Ti	0.4(1)	2.2(3)
Fe	98.5(8)	95.4(7)
Total	100.0	100.0

Note: Numbers in parentheses indicate standard deviation in the last digit.
Concentration of Au was subtracted from raw EPMA data.

Supplementary Table 3 | Summary of fitting parameters

	Fe		$Fe_{80}S_{20}$ 10 GPa and higher		$Fe_{57}S_{43}$ 6.6 GPa and higher	
	This study	This study + ref. 25	This study	This study + ref. 26	This study	This study + ref. 27
K_{S0} (GPa)	101.5(3.3)	110.0(1.8)	84.4(31.1)	93.1(3.0)	34.3(6.4)	31.4(10.5)
K_S'	6.1(1)	4.91(0)	5.6(3)	4.75(1)	6.5(2)	7.2(5)
ρ_0 (g cm^{-3})	6.98[a]	6.98[a]	6.2	6.2	4.7	4.7

Values for $Fe_{80}S_{20}$ and $Fe_{57}S_{43}$ are uncertain but can be used for the calculation of V_P.
[a]Ref. 29

Supplementary References

1. Terasaki, H. *et al.* Pressure and composition effects on sound velocity and density of core‐forming liquids: Implication to core compositions of terrestrial planets. *J. Geophys. Res. Planets,* **124**, 2272– 2293 (2019).
2. Kawaguchi, S. I. *et al.* Sound velocity of liquid Fe-Ni-S at high pressure. *J. Geophys. Res. Solid Earth* **122**, 3624–3634 (2017).
3. Jing, Z. *et al.* Sound velocity of Fe-S liquids at high pressure: Implications for the Moon's molten outer core. *Earth Planet. Sci. Lett.* **396**, 78–87 (2014).
4. Nishida, K. *et al.* Towards a consensus on the pressure and composition dependence of sound velocity in the liquid Fe–S system. *Phys. Earth Planet. Inter.* **257**, 230–239 (2016).
5. Anderson, W. W. & Ahrens, T. J. An equation of state for liquid iron and implications for the Earth's core. *J. Geophys. Res.* **99**, 4273–4284 (1994).
6. Sanloup, C. *et al.* Structural changes in liquid Fe at high pressures and high temperatures from Synchrotron X-ray diffraction. *Europhys. Lett.* **52**, 151–157 (2000).
7. Lin, J. F. *et al.* Magnetic transition and sound velocities of Fe_3S at high pressure: Implications for Earth and planetary cores. *Earth Planet. Sci. Lett.* **226**, 33–40 (2004).
8. Chen, B., Gao, L., Funakoshi, K. & Li, J. Thermal expansion of iron-rich alloys and implications for the Earth's core. *Proc. Natl. Acad. Sci. USA*. **104**, 9162–9167 (2007).
9. Zhang, J. & Guyot, F. Thermal equation of state of iron and $Fe_{0.91}Si_{0.09}$. *Phys. Chem. Miner.* **26**, 206–211 (1999).
10. Tsujino, N. *et al.* Equation of state of γ-Fe: Reference density for planetary cores. *Earth Planet. Sci. Lett.* **375**, 244–253 (2013).
11. Nasch, P. M., Manghnani, M. H. & Secco, R. A. Anomalous behavior of sound velocity and attenuation in liquid Fe-Ni-S. *Science* **277**, 219–221 (1997).
12. Clark, G. N. I., Hura, G. L., Teixeira, J., Soper, A. K. & Head-Gordon, T. Small-angle scattering and the structure of ambient liquid water. *Proc. Natl. Acad. Sci. USA* **107**, 14003–14007 (2010).
13. Takimoto, K. & Endo, H. Sound velocity of liquid se-te mixture. *Phys. Chem. Liq.* **12**, 141–150 (1982).
14. Kajihara, Y. *et al.* Static and dynamical inhomogeneity at liquid-liquid phase transition of Se-Te mixtures. *EPJ Web Conf.* **15**, 02002 (2011).

15. Boehler, R., von Bargen. N. & Chopelas, A., Melting, thermal expansion, and phase transitions of iron at high pressures. *J. Geophys. Res.* **95**, 21731–21736 (1990).
16. Shen, G., Mao, H. K. & Hemley, R. J., Melting and crystal structure of iron at high pressures and temperatures. *Geophys. Res. Lett.* **25**, 373–376 (1998).
17. Ryzhenko, B. & Kennedy, G. C., The effect of pressure on the eutectic in the system Fe–FeS. *Am. J. Sci.* **273**, 803–810 (1973).
18. Sharp, W. E., Melting curves of sphalerite, galena, and pyrrhotite and the decomposition curve of pyrite between 30 and 65 kilobars. *J. Geophys. Res.* **74**, 1645–1652 (1969).
19. Boehler, R., Melting of the Fe-FeO and the Fe-FeS systems at high pressure: Constraints on core temperatures. *Earth Planet. Sci. Lett.* **111**, 217–227 (1992).
20. Anderson, W. W., & Ahrens, T. J., Shock temperature and melting in iron sulfides at core pressures, *J. Geophys. Res.*, **101**, 5627–5642 (1996).
21. Stewart, A. J., Schmidt, M. W., van Westrenen, W. & Liebske, C. Mars: a new core-crystallization regime, *Science* **316**, 1323–1325 (2007).
22. Usselman, T. M., Experimental approach to the state of the core; Part I, the liquidus relations of the Fe-rich portion of the Fe-Ni-S system from 30 to 100 kb. *Am. J. Sci.* **275**, 278–290 (1975)
23. Rivoldini, A., Van Hoolst, T., Verhoeven, O., Mocquet, A. & Dehant, V. Geodesy constraints on the interior structure and composition of Mars. *Icarus* **213**, 451–472 (2011).
24. Khan, A. *et al.* A geophysical perspective on the bulk composition of Mars. *J. Geophys. Res. Planets,* **123**, 575–611 (2018).
25. Nguyen, J. H. & Holmes, N. C. Melting of iron at the physical conditions of the Earth's core. *Nature* **427**, 339–342 (2004).
26. Huang, H. *et al*. Measurements of sound velocity of liquid Fe-11.8 wt % S up to 211.4 GPa and 6,150 K. *J. Geophys. Res. Solid Earth* **123**, 4730–4739 (2018).
27. Shaner, J. W., Hixson, R. S., Winkler, M. A., Boness, D. A. & Brown, J. M. Birch's law for fluid metals. In *Shock Waves in Condensed Matter* (eds. Schmidt, S.C. & Holmes, N.C.) 135-138 (Elsevier, 1988).
28. Nasch, P. M., Manghnani, M. H. & Secco, R. A. Sound velocity measurements in liquid iron by ultrasonic interferometry. *J. Geophys. Res.* 99, 4285–4291 (1994).
29. Nasch, P. M. & Manghnani, M. H. Molar volume, thermal expansion, and bulk modulus in liquid Fe-Ni alloys at 1 bar: Evidence for magnetic anomalies? in *Properties of Earth and Planetary Materials at High Pressure and Temperature* (eds. Manghnani, M. H. & Yagi, T.) 307–317 (AGU, 1998).

30. Guillermet, A. F., Hillert, M., Jansson, B. & Sundman, B. An assessment of the Fe-S system using a two-sublattice model for the liquid phase. *Metall. Trans. B* **12**, 745–754 (1981).
31. Buono, A. S. & Walker, D. The Fe-rich liquidus in the Fe-FeS system from 1bar to 10GPa. *Geochim. Cosmochim. Acta* **75**, 2072–2087 (2011).
32. Marqués, M., González, L. E. & González, D. J. Pressure-induced changes in structural and dynamic properties of liquid Fe close to the melting line. An ab initio study. *J. Phys. Condens. Matter* **28**, 075101 (2016).
33. Ichikawa, H., Tsuchiya, T. & Tange, Y. The P-V-T equation of state and thermodynamic properties of liquid iron. *J. Geophys. Res. Solid Earth* **119**, 240–252 (2014).
34. Brown, J. M. & McQueen, R. G. Phase transitions, Grüneisen parameter, and elasticity for shocked iron between 77 GPa and 400 GPa. *J. Geophys. Res.* **91,** 7485–7494 (1986).
35. Umemoto, K. *et al.* Liquid iron-sulfur alloys at outer core conditions by first-principles calculations. *Geophys. Res. Lett.* **41,** 6712–6717 (2014).
36. Liu, L.-G., & Bassett, W. A., The melting of iron up to 200 kbar, *J. Geophys. Res.*, **80**, 3777–3782 (1975).
37. Anzellini, C. *et al.* Melting of iron at Earth's inner core boundary based on fast X-ray diffraction. *Science* **340**, 464–466 (2013).
38. Williams, Q. & Jeanloz, R., Melting relations in the iron-sulfur system at ultra-high pressures: Implications for the thermal state of the Earth. *J. Geophys. Res.* **95**, 19299–19310 (1990).
